# An Improved Acoustic Diffusion Equation Model for Long-Channel Underground Spaces

**DOI:** 10.3390/s23187738

**Published:** 2023-09-07

**Authors:** Chao Mou, Qiliang Yang, Jianchun Xing, Tao Chen, Rongwei Zou

**Affiliations:** College of Defense Engineering, Army Engineering University of PLA, Nanjing 210001, China; mocolocm@foxmail.com (C.M.); xjc@893.com.cn (J.X.); ct2268662256@163.com (T.C.); zrwlyf@163.com (R.Z.)

**Keywords:** acoustic diffusion equation model, long-channel type, underground space, compensation

## Abstract

The acoustic diffusion equation model has been widely applied in various scenarios, but a larger prediction error exists when applied to underground spaces, showing a significantly lower characteristic of the sound pressure level in the later stage compared to field tests since underground spaces have a more closed acoustic environment. Therefore, we analyze the characteristics of underground spaces differentiating from aboveground spaces when applying the model and propose an improved model from the perspective of energy balance. The energy neglected in the calculation of the acoustic diffusion equation model is compensated in long channel underground spaces named “acoustic escape compensation”. A simulation and two field experiments are conducted to verify the effectiveness of the proposed compensation strategy in long-channel underground spaces. The mean square error is used to evaluate the differences between the classical model and the improved model, which shows a numerical improvement of 1.3 in the underground field test. The results show that the improved model is more suitable for describing underground spaces. The proposed strategy provides an effective extension of the acoustic diffusion equation model to solve the problem of sound field prediction and management in underground spaces.

## 1. Introduction

As aboveground space becomes increasingly crowded, underground space plays a growing role in modern society. The development and utilization of underground spaces, such as underground shopping malls, underground corridors, and underground parking lots, have become increasingly significant; these spaces can effectively supplement the crowded aboveground spaces in modern cities and alleviate surface traffic and commercial space congestion.

With research on smart cities increasing in recent years [1], the development and management of underground spaces have become increasingly strategic [2,3]. The characterization of various physical fields in underground space has attracted increasing attention to the careful management of underground space. As noteworthy physical fields in underground space, acoustic fields have attracted attention from relevant scholars; for example, some have investigated the impact of the acoustic environment in underground spaces on safety [4] and the reference factors of acoustic field characteristics in underground spaces for design purposes [5]. Elucidating the sound field characteristics of underground spaces is thus important for the intelligent operation and maintenance management of underground spaces.

There has been some research regarding the propagation characteristics of acoustic fields in underground spaces. Kang Jian [6,7,8,9,10,11] conducted in-depth research on the acoustic characteristics of long enclosed spaces (a type of underground space) in the 1990s, which focused on explaining the unsuitability of classical room acoustic theory in underground spaces [8] and the proposal of a sound field calculation method based on statistical theory. Later, scholars in the field of underground space acoustic fields mainly focused on noise reduction or noise suppression in highway tunnels [12,13,14]. Notably, there has been relatively little research on the laws governing the propagation of acoustic fields in underground spaces.

There has been extensive research in the field of room acoustic prediction, with early research mainly focusing on geometric and statistical acoustics. During this period, Sabine proposed the classic Sabine reverberation formula [15], which is thought to have pioneered the study of room acoustic research [16]. Subsequently, scholars conducted research on the characteristics of sound waves in the field. Ma Dayou revised the formula for the normal number of frequencies in sound waves and gave a reverberation solution for a rectangular room with a uniform nondamping interface [15]. Later, scholars made positive contributions to acoustic theory based on the geometric characteristics of sound waves. Ollendorff [17] first proposed the idea of diffusion theory in room acoustics, which has been further improved and studied by many scholars and widely applied in the field of acoustic prediction [18].

When applying the acoustic diffusion equation (ADE) model to underground space, the predicted sound pressure level (SPL) attenuation is often not sufficiently accurate, with the value being lower at the later stage [19]. However, under the same conditions aboveground, the ADE model has obvious advantages in computing speed compared with other acoustic prediction methods [18,19]. How to further improve the prediction accuracy while exploiting this advantageous computational efficiency has become an important research question. Therefore, based on theoretical analysis and experimental verification, a strategy of “acoustic escape compensation” (AEC) for long-channel underground space is proposed here; this compensation strategy improves the accuracy significantly at the later stage of the diffusion equation model in underground space.

The rest of this paper is organized as follows: Part two describes the basic theory of the ADE model, which introduces the derivation and development of the ADE model. Part three describes the AEC method proposed in this paper. From the perspective of energy balance, the classical model is improved. Part four shows the simulated comparison between the proposed compensation strategy and the model without compensation. The simulation results meet the expectation, i.e., the later stage of SPL shows a noticeable increase. Part five describes two field experiments to verify the classical and improved models, using SPL as a parameter to compare with the results. The first experiment is conducted to verify the effectiveness of the ADE model in long-channel spaces, while the second experiment is conducted to verify the effectiveness of the improved model applied to long-channel underground spaces. The sixth part concludes the paper and discusses the improved model.

## 2. Basic Theory of the ADE Model

The ADE model has a wide range of applications in the field of acoustics. Olledorff [17] first proposed the idea of acoustic diffusion theory, which assumes that each sound particle propagates at the speed of sound and forms a diffuse sound field in space. Picaut [20] and others proposed a mathematical model of the diffuse sound field and then simulated and verified the applicability of the ADE model in a regular long space by deriving the method of calculating the model [21]. Based on the simulated results, the model calculation method proposed by Picaut conforms to the basic assumptions of the space sound field, and this work represents a considerable contribution to the further study of the model. Valeau [22] and others further studied and discussed the boundary conditions of the ADE model and clarified the calculation method of the acoustic source term q, in turn making the calculation of the ADE model clearer. Based on the ADE model, Billon et al. [23] introduced the concept of environmental attenuation of the diffuse sound field, further optimizing the model accuracy, and then they modified the boundary conditions of the model and applied them to the prediction of reverberation time [24]. At the same year, Jing et al. [25] visualized the sound energy flow based on the ADE model and proposed a modified boundary condition, which combines the advantages of Sabine and Eyring boundary conditions [26]. Escolano et al. [27] discussed the limitation of the ADE model, pointed out that more experiments are needed to compare the accuracy of the model in more complex indoor Spaces. Billon et al. [28] applied the ADE model in an industrial space, which further verified the applicability of the ADE model and demonstrated an excellent calculation load compared with geometric acoustic calculation methods such as the ray tracing method. Navarro [29] and others expanded the ADE model based on principles of the radiation model, but the assumptions of the expanded models were relatively strict while still failing to solve the issues of the ADE model in predicting early attenuation and having a higher calculation load than that of the classical model. Moreover, they studied the influence of the scattering and absorption coefficients on homogeneous room simulation [30]. Later, scholars further studied the diffusion equation model, verified its applicability in various room spaces [31], and introduced the idea of transmission coefficient *τ* in coupled spaces. This idea was further applied to coupled spaces by optimizing the boundary conditions [32]. Foy et al. [33] extended the ADE model by modifying the absorption and scattering coefficients. This extension improved the prediction accuracy, but the more complicated expression increased the computational load, and its application is limited.

**Figure 1:**
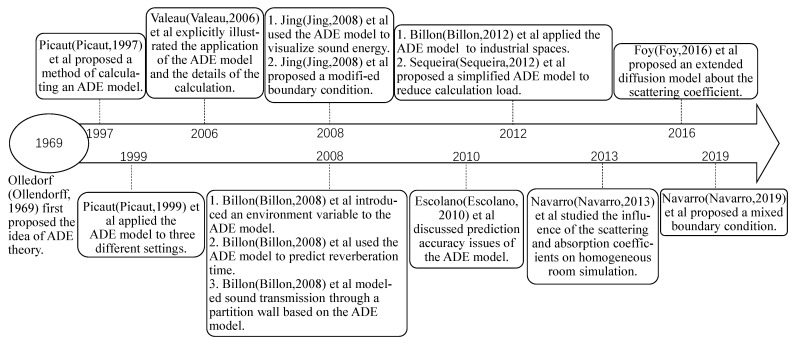
Timeline of the development of the ADE model. The references mentioned in the figure are [17,20,21,22,23,24,25,26,27,28,30,32,33,34,35].

Generally, the ADE model provides an efficient solution for room acoustic simulation, prediction, and management. The history of developing the ADE model is shown in Figure 1. The ADE model has significantly progressed in the past 20 years.

Acoustic diffusion theory is an extension of Fick’s law, namely,

(1) $$J=-D\cdot grad\left(\omega \left(r,t\right)\right)$$

where *J* represents the energy flux, ***r*** and *t* represent the position vector and time, respectively, *D* represents the diffusion coefficient, and *grad*(ω(***r***,*t*)) represents the gradient of the energy density ω. The classical form of the acoustic diffusion equation is:

(2) $$\frac{\partial \omega \left(r,t\right)}{\partial t}-D\nabla^{2}\omega \left(r,t\right)=q\left(r,t\right)$$

(3) $$-D\frac{\partial \omega \left(r,t\right)}{\partial n}=A_{X}\left(r,\alpha \right)c\omega \left(r,t\right)$$

Equation (3) is the boundary condition for solving the partial differential Equation (2). In Equations (2) and (3), ▽^2^ is the Laplace operator, ω(***r***,*t*) is the sound energy flow density at time *t* at position ***r***, *q*(***r***,*t*) is the sound source term, ***n*** is the normal vector of the boundary, *A*_X_(***r***,*α*) is the boundary absorption condition, and the Sabine boundary condition is used in this article. According to the text previously presented, two assumptions regarding the governing equation are needed:

(1) The sound particles diffuse adequately in the medium, meaning that the reflections on the reflection objects are totally diffuse, and the sound energy density is distributed uniformly in homogeneous dimensions across the room space.
(2) Energy flux changes are much lower than when sound crosses a mean free path. This property is sometimes called temporal broadening.

Thus, the ADE model can predict the SPL and reverberation time. Additionally, there are two principles worth noting:

(i) Uniform sound energy density does not equal uniform sound pressure or sound pressure level. Specifically, sound energy includes kinetic energy and potential energy, while sound pressure only represents potential energy [36];
(ii) The diffusion model does not inherently take the frequency information into account [31].

Compared with the ray tracing method and the simulation method based on wave theory, the ADE model has the advantages of low computational load [34] and relatively high prediction accuracy. It has good applicability in the prediction of room acoustic fields and has strong application prospects, such as for digital twins and on-site monitoring in highly interactive environments. However, it is worth noting that the ADE model is built on the basis of energy conservation. The first-order approximation of energy conservation is used in the room acoustics calculation of the classical model. Thus, only the absorption of the diffusion space interior and the space wall is considered; importantly, the energy dissipation after the sound energy transmission outside the wall is not considered, which causes prediction errors, especially when the transmission coefficient is large. Here, the ADE model is improved by taking the energy dissipation of the sound field after wall transmission into consideration to make the ADE model more suitable for describing underground spaces.

## 3. Improved ADE Model

In this section, from the perspective of energy balance, the sound field characteristics of underground spaces are analyzed. We point out the energy overlooked when applying the classical model to underground space calculations. Then, combining the research of relevant scholars on this part of the method’s energy, the AEC strategy is proposed and explained, and it is deduced in three steps.

### 3.1. Analysis of Acoustic Characteristics in Underground Space

Kang’s [37] team conducted in-depth research on the acoustic characteristics of underground spaces. In their experimental analysis of the acoustic environment in underground spaces [38], they mentioned that the reflection and attenuation time of acoustic fields in underground spaces is longer, and the SPL also attenuates more slowly; these findings represent differences in the acoustic characteristics of underground spaces compared to conventional aboveground spaces. The main reason for these differences is that the diffusion of acoustic fields in underground spaces is not as thorough as that in ground spaces since underground spaces are more closed. Classical theories are not suitable for describing underground spaces [10].

**Figure 2:**
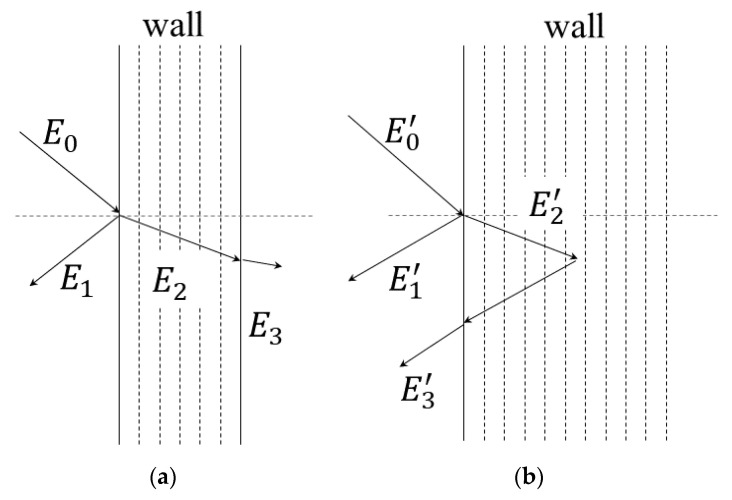
Schematic diagram of sound energy absorption and reflection of walls in aboveground and underground spaces. *E*_3_ is the energy “escaped” in the calculation. (**a**) Ordinary aboveground room space; (**b**) underground room space.

The energy relationship between conventional room spaces above the ground and underground spaces can be analyzed. Figure 2a shows the energy relationship diagram of a wall in the conventional aboveground room space, where *E*_0_ represents the incident energy, *E*_1_ represents the energy reflected by the wall surface, *E*_2_ represents the energy absorbed by the wall, and *E*_3_ represents the energy transmitted through the wall. It can be obtained that. In the calculation of classical models, *E*_3_ is often neglected. Figure 2b shows the energy relationship diagram of underground space walls, where $E_{0}^{\prime }$ represents the incident energy, $E_{1}^{\prime }$ represents the energy reflected by the wall surface, $E_{2}^{\prime }$ represents the energy absorbed by the wall, and $E_{3}^{\prime }$ represents the energy that cannot be absorbed by the wall. Similarly, it can be obtained that $E_{0}^{\prime }=E_{1}^{\prime }+E_{2}^{\prime }+E_{3}^{\prime }$ .

Conventional room space walls have a certain thickness, and some of the energy that cannot be absorbed by the walls is transmitted and “escaped” into the outside space. However, in underground spaces, due to the infinite (compared to wavelength) thickness of the walls, in most cases, some of the energy that cannot be absorbed by the walls continues to be scattered back into the space through reflection. In other words, the sound transmission loss in underground spaces is theoretically infinite. In calculations, the corresponding material absorption coefficient of the conventional room space is generally used, which leads to prediction errors and is the reason why underground spaces have a longer decay time compared to conventional aboveground space. Accordingly, a compensation method is necessary to obtain more accurate predictions.

### 3.2. Improved ADE Model

**Figure 3:**
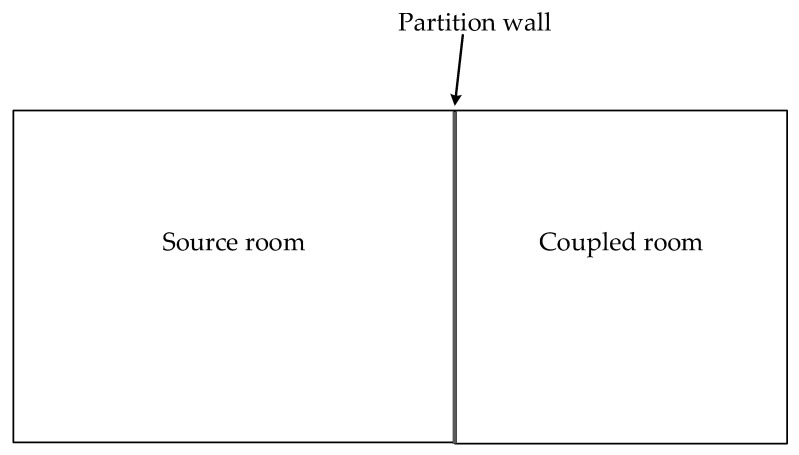
Diagram of a coupled room system with a partition wall: source room (*V*_1_, *S*_1_, $\alpha_{s}$); coupled room (*V*_2_, *S*_2_, $\alpha_{a}$). The partition wall is defined by its transmission loss R and surface *S*_12_.

There are two kinds of boundary conditions considered in the classical ADE model, namely, total reflection and transmission. In underground spaces, in addition to the previously described differences, there is no total reflection condition, and transmission is also different. Therefore, it is necessary to establish a new energy balance relationship specific to underground spaces. It is built in three steps as follows:

(1) Valeau et al. [22] described the sound energy flux after transmission when analyzing and studying the ADE model in detail with the following equation:

(4) $$J_{in}=\frac{\tau c\omega_{1}}{4}$$

where *J_in_* is the rate of sound energy flowing into the second room, *ω*_1_ is the sound energy density of the first room (sound source), *τ* is the transmission coefficient, and *c* is the speed of sound. Following the previous analysis, the flow of sound energy returns to the underground space for further propagation here;

(2) Billon et al. [35] applied statistical theory to the study of sound transmission. A diagram from this study is shown in Figure 3 to illustrate the impact of the transmission coefficient in a coupled room.

The relationship between ω_1_ and ω_2_ in the source room and coupled room, respectively, was expanded as follows:

(5) $$\alpha_{a}S_{2}w_{2}+\alpha_{12}S_{12}w_{2}=\tau S_{12}w_{1}$$

where *α_a_* represents the absorption coefficient of the sound source space, *S*_2_ represents the surface area of the second room, *α*_12_ represents the sound absorption coefficient of the partition wall, and *S*_12_ represents the area of the partition wall. It can be obtained that *S*_2_ = *S*_12_ and *α_a_* = *α*_12_ under the conditions of underground spaces in most cases. Therefore, the following equation is obtained:

(6) $$\omega_{2}=\frac{\tau }{2\alpha }\omega_{1}$$

(3) Combined with the research of Billon et al. [23] on acoustic field environment variables, the energy balance in the room can be obtained by integrating Equation (7) over the volume *V*:

(7) $$\int_{V}\frac{\partial \omega \left(r,t\right)}{\partial t}dV_{r}-\int_{V}D\nabla^{2}\omega \left(r,t\right)dV_{r}=\int_{V}P\left(r,t\right)dV_{r}+\int_{V}c\frac{\tau }{2\alpha }\omega \left(r,t\right)dV_{r}$$

Consequently, an improved ADE model more suitable for underground space can be obtained:

(8) $$\frac{\partial w\left(r,t\right)}{\partial t}-D\nabla^{2}w\left(r,t\right)-c\frac{\tau }{2\alpha }w\left(r,t\right)=q\left(r,t\right)$$

where *q*(***r***,*t*) represents the sound source (note: the sound source discussed in this paper is omnidirectional), *c* represents the propagation speed of sound in the medium, 343 m/s in this paper, and *D* is the diffusion coefficient. In addition, $D=\frac{\lambda c}{3}$, where *λ* is the average free path and $\lambda =\frac{4V}{S_{t}}$. *V* is the volume of the space, and *S_t_* is the surface area of the room. In the application of boundary conditions, the Sabine boundary condition is used. That is, $A_{X}\left(r,\alpha \right)=\frac{\alpha }{4}$ is taken in Equation (3).

After obtaining the sound energy density distribution of the space sound field, the SPL is calculated.

(9) $$\mathrm{SPL}=10\mathrm{lg}\frac{\omega \left(r,t\right)\rho c^{2}}{P_{ref}^{2}}$$

The distribution of the SPL along the one-dimensional depth can be obtained, where *P_ref_* is the reference value of sound pressure, taken as 2 × 10^−5^ Pa, and *ρ* is the density of air. The numerical value is 1.2. Then, the one-dimensional attenuation of SPL along the depth at the height of the sound source can be obtained.

## 4. Simulation Verification

Simulation experiments have obvious advantages compared to field experiments for this type of research since the structure of walls cannot be shifted easily in underground spaces. In this section, a simulation experiment is conducted to verify the AEC strategy. Based on the assumptions in Section 2, two simulation objectives can be anticipated:

(1) The energy density is almost uniform. The simulated results for the sound energy density should be in line with the previous assumption of uniformity;
(2) The SPL values using the AEC model at the later stage are apparently higher compared to the ADE model.

The simulation experiment will be invalid if the first simulation objectives cannot be achieved. In contrast, the simulation is effective if the energy density is uniform. In addition, the AEC strategy can be validated for its effectiveness in simulation experiments based on the second simulation objective.

### 4.1. Simulation Conditions and Parameter Settings

**Figure 4:**
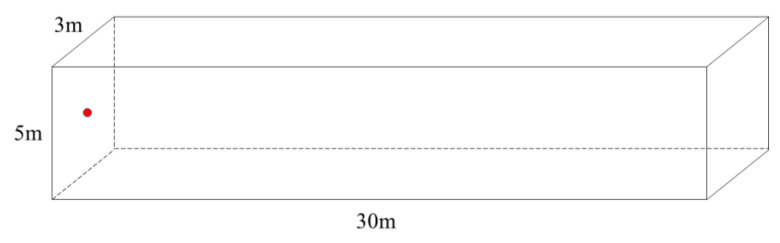
Diagram of the simulated underground space. The red point represents the position of the source.

To verify the ideas proposed above, we combine the fields frequently involved in our team’s research. We take a 3 m wide, 5 m high, and 30 m deep tunnel-type underground space as an example, as shown in Figure 4, and select three different transmission coefficients with relatively large differences to compare the outcomes. The wall on the left is faced with concrete, and SPL attenuation is selected as the outcome for evaluation. The proposed AEC strategy is validated in MATLAB v.R2022b (MathWorks, Natick, MA, USA) simulation software. The omnidirectional sound source is at the center of the left wall. The rightmost side is an opening. The finite difference method is adopted to calculate the SPL attenuation along the length (depth) direction of the sound source under one-dimensional conditions.

MATLAB is a powerful mathematical software. It can be used in various fields, such as data analysis, deep learning, and signal processing. The pdepe function is a built-in function in MATLAB that is used for solving partial differential equations. This function uses the finite difference method to solve the partial differential equations. The diffusion equation is a four-dimensional equation (*x*, *y*, *z,* and *t*). To obtain the variation of the sound energy density along the length direction at the height of the sound source, two of these dimensions need to remain unchanged, i.e., *y* and *z*. Thus, the relationship between the sound energy density ω, time *t*, and length *x* can be obtained with the help of the pdepe function in MATLAB. The hardware environment of the simulation running is 11th Gen IntelI CITM) i7-11800H @ 2.30GHz.

By consulting previously collected data [39] (pp. 540–549), the sound absorption coefficient and the transmission loss can be obtained. The sound absorption coefficient of concrete is *α* = 0.02 in common frequency bands, so we use this value in the simulation. The transmission loss varies from 34 dB to 52 dB under different building structural conditions. For a more obvious display of the comparison, we take the sound reduction index as one of three values, 35 dB, 40 dB, or 50 dB, i.e., *τ* ≈ 3.2 × 10^−4^, *τ* = 10^−4^, or *τ* = 10^−5^, respectively, to compare the SPL attenuation under three different transmission coefficient conditions of the AEC model with that of the classical model.

By estimation, the sound energy density reaches a steady state within approximately 0.4 s, so the period between 0 and 0.4 s is simulated. The 100 × 100 grids are considered. The room space area is calculated based on the length of the side with the opening. The values of *D* and *λ* can be obtained. The boundary condition is determined using the Sabine absorption condition. By using MATLAB partial differential equation calculation tools, the energy density variation along the length (depth) can be obtained.

### 4.2. Simulation Results

**Figure 5:**
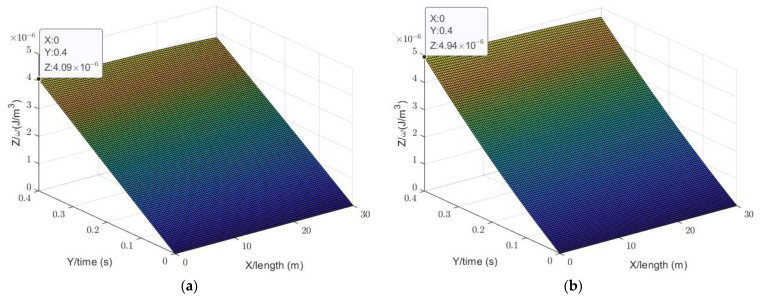

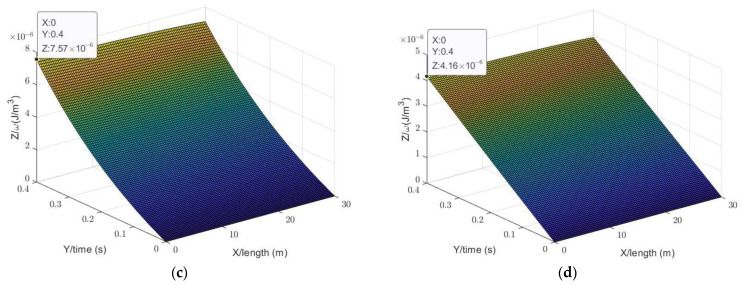
The sound energy density distribution at the height of the sound source along the length with different *τ* values: (**a**) sound energy density variation in the classical model; (**b**) sound energy density variation in the AEC model when *τ* = 10^−4^; (**c**) sound energy density variation in the AEC model when *τ* ≈ 3.2 × 10^−4^; and (**d**) sound energy density variation in the AEC model when *τ* = 10^−5^.

Figure 5a shows the relationship of the sound energy density, time, and distance at the height of the sound source along the depth under one-dimensional conditions without using the AEC model. Figure 5b shows the AEC simulated results for *τ* = 10^−4^. Figure 5c shows the AEC simulated results for *τ* ≈ 3.2 × 10^−4^. Figure 5d shows the AEC simulated results for *τ* = 10^−5^.

The sound energy density in the simulation results shows a uniform characteristic. It meets the first anticipated objective. Therefore, it can be concluded that the simulation is valid, although there are minor changes from the source point to the end. The larger the transmission coefficient is, the greater the impact on the sound energy density in the space when using the AEC model. At the same time, when the transmission coefficient is as small as 10^−5^, the compensatory effect offered by the AEC strategy relative to the classical model is very weak.

### 4.3. SPL Attenuation Analysis

**Figure 6:**
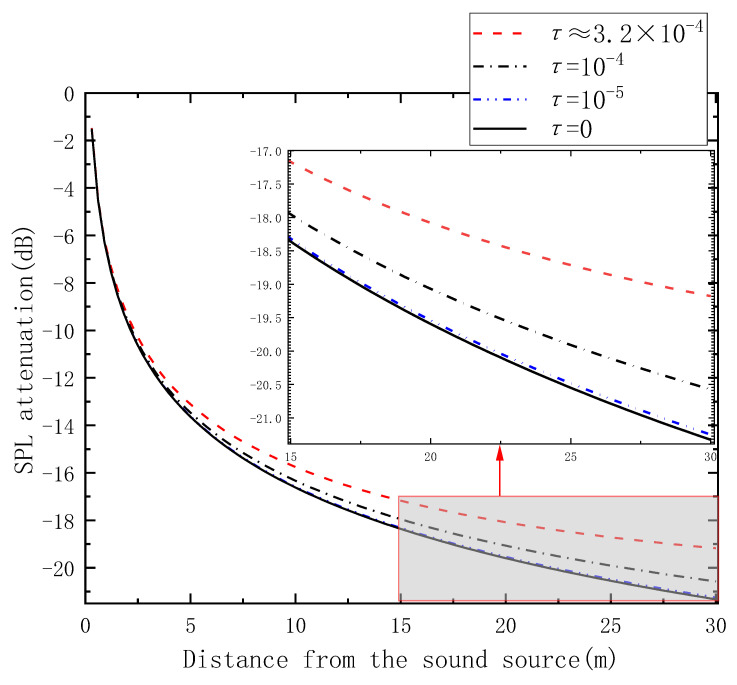
SPL attenuation under different compensation conditions.

The SPL characteristics of the sound source at the height of the sound source along the length dimension are shown in Figure 6. Compared to the classical model, SPL attenuation shows differences of increasing magnitude with increasing distance. The larger the value of transmission coefficient *τ* is, the greater the difference in SPL attenuation. Table 1 lists the SPL attenuation values (with two significant digits after the decimal point) for four conditions at distances of 10 m, 20 m, and 30 m from the sound source. Based on the data in the table, it can be concluded that at a distance of 30 m, i.e., at the end of the space, the SPL attenuation difference between the AEC model at *τ* ≈ 3.2 × 10^−4^ and the classical model is approximately 2.2 dB.

However, the difference is very small when the transmission coefficient is small enough. The AEC model is effective in improving the ADE model when applied to long-channel underground spaces in simulations.

According to the simulation results, at the transmission coefficient *τ* ≈ 3.2 × 10^−4^, the difference in the sound energy density between the AEC model and the classical model is approximately 3.5 given the same situation, which represents an improvement in modeling underground spaces relative to the classical ADE model. The AEC model can be applied to long-channel underground spaces well, allowing it to provide guidance to optimize the selection of materials for walls in underground spaces and other features of the underground space acoustic environment.

## 5. Field Experiments

In this section, two field experiments are described; one field experiment shows the applicability of the ADE model in aboveground long-channel type spaces, and another shows the applicability of the AEC model in underground long-channel type spaces. The two field experiments have similar environmental parameters, such that the applicability of both models can be verified. The SPL at different positions along the length of the sound source was recorded as the evaluation parameter in both experiments. Due to the limitation of site conditions, the structure of the walls in the underground space could not be altered easily, so the measurements are used in the classical model and AEC model for comparison. Finally, the mean squared errors of the AEC model and classical model relative to the real values are compared to evaluate the benefit of the AEC model.

### 5.1. Experimental Environment

First, we select a corridor inside a surface building and a corridor of an underground space.

**Figure 7:**
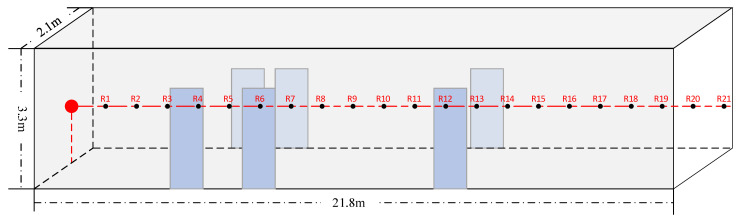
Schematic diagram of the aboveground space experimental environment. The sound source is located at the red point, and it is 1.3 m high in the middle of the width. R1 to R21 are the positions of the receivers and are in line with the sound source at the height of 1 m intervals between each two receiving positions. The six blue rectangles represent six iron gates.

The corridor inside the aboveground building is 21.8 m long, 2.1 m wide, and 3.3 m high, as shown in Figure 7. There are 1.3 m high marble tiles on both walls and six iron gates along the length. The floor is fully covered with marble tiles. There are four 50 cm × 100 cm fiberglass boards near the ceiling. By consulting relevant data [40], a mean absorption coefficient of 0.02 for the space was selected since the iron gates and marble tiles have almost the same absorption coefficients as the cement wall in most frequency ranges. The impact of several small decorative panels on the calculation can be ignored.

**Figure 8:**
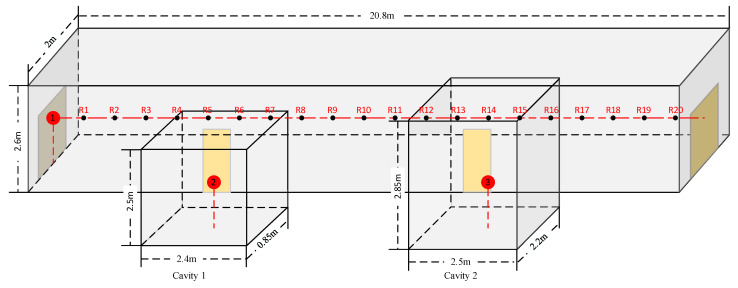
Schematic diagram of the underground space experimental environment. Red Point 1 represents the location of the sound source, which is 1.3 m high and in the middle of the width. Red Points 2 and 3 represent the SPL receiving points of the two cavities, respectively. R1 to R20 are 20 SPL receiving points separated by one meter. All of them are in line with the height of the sound source. The two yellow rectangles on the side wall are solid wood doors.

The corridor in the underground spaces is 20.8 m long, 2.6 m high, and 2 m wide. The two experimental environments had very similar parameters. Therefore, it is valid to apply the ADE model and evaluate the improvements of the AEC model in underground space after verification in aboveground space. There are two cavities on one side of the corridor, and their distances from the left and right ends are 6 m and 7.1 m, respectively. The two cavities have different sizes, as shown in Figure 8. There is a wooden door between the cavities and the corridor. The wooden door is 76 cm × 190 cm and is made of solid wood. Based on previously collected data, the transmission loss of a solid wooden door is 35 dB under standard conditions [41], that is, the transmission coefficient *τ* ≈ 3.2 × 10^−4^. There are two iron gates at both ends of the corridor. The gates are 1.3 m × 1.8 m. Thus, the corridor can be regarded as a semi-enclosed space when one side door is closed.

The ground corridor experiment was conducted on 1 June 2023. The photos and results of the experiment are presented in Section 5.2. The underground field tests were conducted on the 1, 2, and 4 of June 2023. No other noise on site interfered while the tests were ongoing.

An AWA5510A standard dodecahedron omnidirectional sound source is used for acoustic excitation and driven by an AHAI (Type 2042) power amplifier. An AWA (type 5636) microphone, which is in accordance with IEC 61672-1:2013 [42], is used to measure the SPL.

The dodecahedron omnidirectional sound source is positioned 1.3 m above the floor in both experiments, located at the left end of the corridor at red point 1 in Figure 8 in the underground corridor. The AWA microphone is placed at one of the measurement points from R1 to R20 in each test at a height of 1.3 m. There is a 1 m interval between each measurement point. At red points 2 and 3 in the middle of cavity 1 and cavity 2, respectively, a microphone was placed to obtain the SPL of sound traveling through the wooden doors.

**Figure 9:**
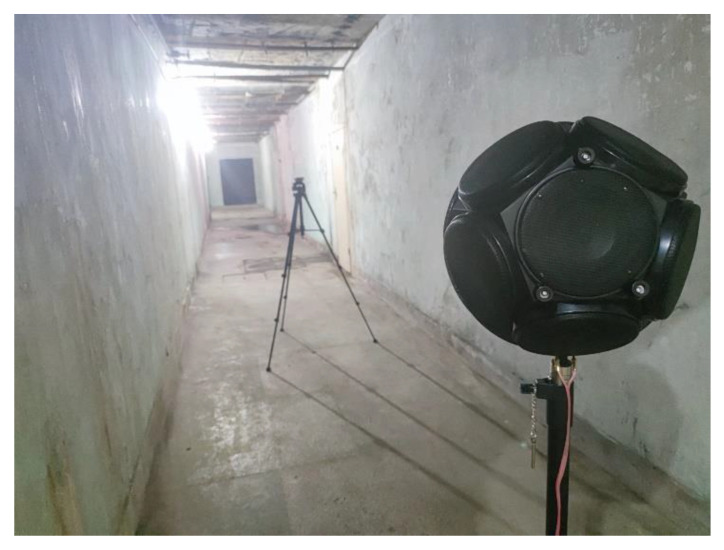
Field test photo in the underground corridor. The dodecahedron omnidirectional sound source is positioned 1.3 m above the floor, and the microphone is 4 m away from the sound source.

To simulate the non-uniform frequency sound generated by equipment in underground spaces, the source signal is set to white noise in aboveground and underground field experiments. White noise refers to the noise whose power spectral density is constant in the whole frequency domain. Ideal white noise has an infinite bandwidth, and thus, its energy is infinite. However, this concept does not occur in the real world. In fact, we often think of finite bandwidth leveling signals as white noise because it makes it easier for us to analyze them mathematically. In our experiments, the frequency bandwidth is from 31.5 Hz to 16 kHz. To eliminate the interference of irrelevant factors, we take the average of the experimental data from three consecutive days in the underground experiment. In each test, 10 SPL data points are recorded after the measurements are stable, and then the average value is taken. Photos of the experimental site are shown in Figure 9. As shown in the figure, there are twelve thin wooden sticks on the ceiling. Their impact on sound field calculations is very small, so their absorption and reflection of sound energy can be ignored. The walls, floor, and ceiling are all plastered with cement. According to the existing frequency-dependent sound absorption coefficient data [39] (pp. 540–549), the sound absorption coefficient is 0.02 in most frequency ranges. Thus, the absorption coefficient can be obtained, i.e., *α* = 0.02. The transmission loss is approximately 40 dB based on the construction method of the interior walls, i.e., *τ* = 10^−4^.

### 5.2. Experimental Results

The SPL data are obtained by an AWA microphone with a 100 dB excitation from the dodecahedron omnidirectional sound source. To avoid the impact of equipment differences, we use the same measurement method multiple times at each receiving point using the same device.

**Figure 10:**
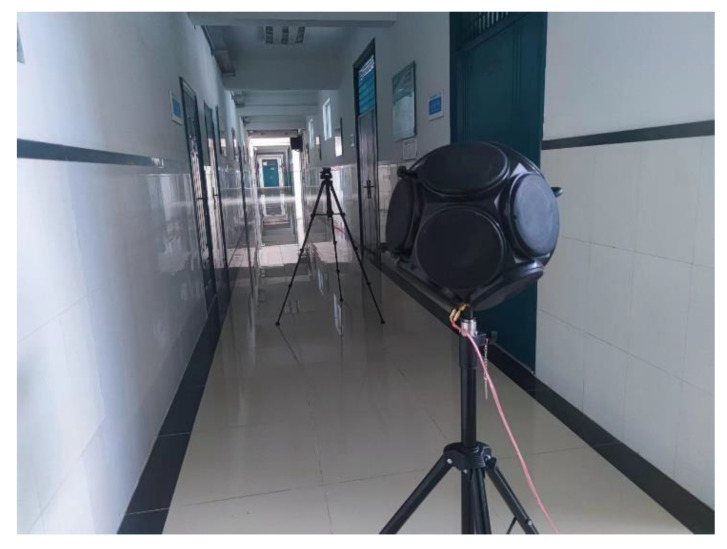
Field test photo in the aboveground corridor. The dodecahedron omnidirectional sound source is placed 1.3 m above the floor, and the microphone is 4 m away from the sound source.

**Figure 11:**
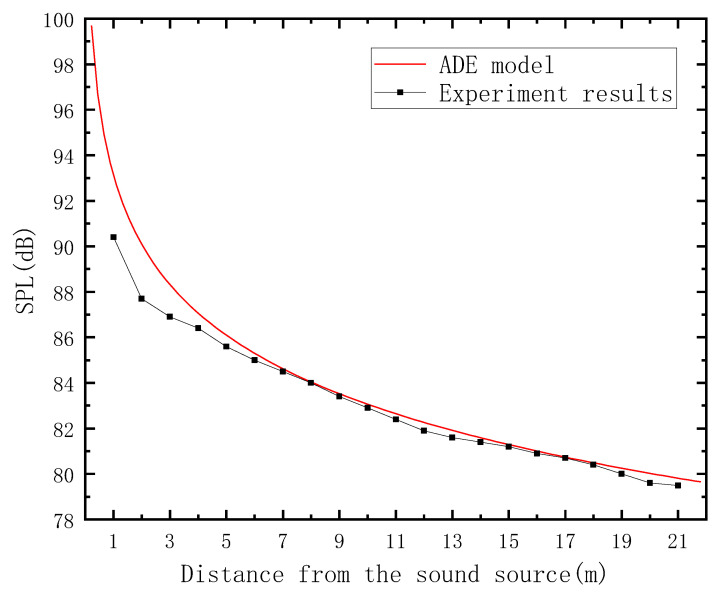
Comparison between the ADE model and experimental results in the aboveground corridor in a building.

The site photo for the aboveground experiment is shown in Figure 10, and the comparison between the experimental and predicted results of the ADE model is shown in Figure 11. The ADE model shows good accordance with the experimental results. Consequently, the applicability of the ADE model in long channel-type spaces is verified.

The recorded data for the underground experiments are shown in Table 2. Data in the table are the average of the 10 data points at that test on that day. The SPL of Red Points 2 and 3 can be compared based on a previously proposed method [35]. In this reference, an equation for the SPL difference was given:

(10) $$L_{1}-L_{2}=R-10\log\frac{S_{12}}{A_{22}}$$

where *L*_1_ and *L*_2_ are the SPL of the first room and second room, respectively, in the ISO 140-4 [43] and EN 12354-1 [44] standards. These standards conform with the standards of the experimental equipment used. *R* is the transmission loss of the partition wall, *S*_12_ represents the area of the partition wall, $A_{22}=\alpha_{a}S_{2}+\alpha_{12}S_{12}$ and $\alpha_{12}=\tau$.

Thus, we can obtain the calculated reference value. The data related to this calculation are shown in Table 3, in which *L*_1_ is the value nearest to that of the solid wood door in the experiment.

As shown in the table, the predicted SPL value of cavity 1 is approximately 57.2 dB; it is approximately 52.0 dB in cavity 2. Compared to the experimental data, the differences are 0.2 dB and 0.9 dB, respectively. The transmission coefficient method shows good accordance with the experiment in cavity 1. The reason why cavity 2 shows a larger difference is that there is a steel water tank in it. The water tank produced significant reflections, resulting in an increase in the measured SPL.

### 5.3. Data Comparison

The ADE model shows good accordance with the experimental results, as shown in Figure 11. However, there is a predictive error in underground spaces; thus, a comparison should be conducted to illustrate the improvement effect of the AEC model.

**Figure 12:**
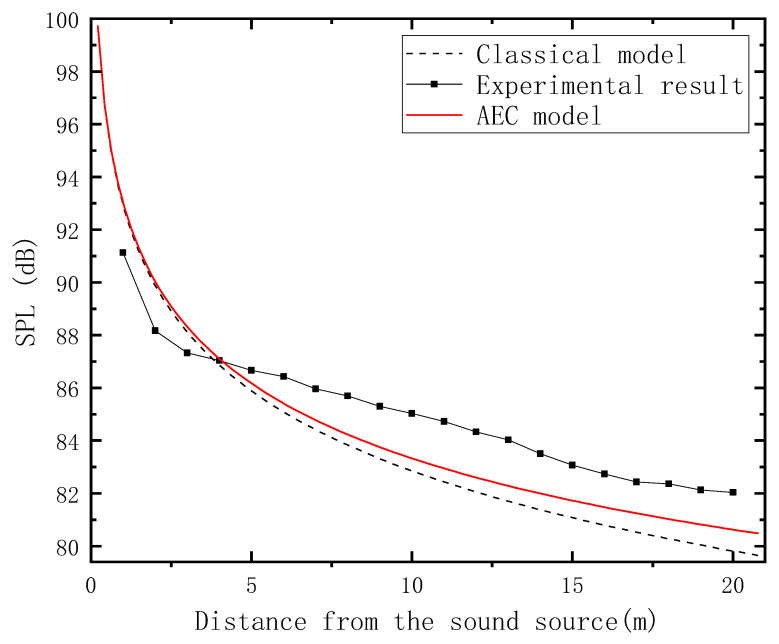
Comparison chart among the experimental results, classical model, and AEC model.

Figure 12 shows the comparison of experimental results with the AEC model and ADE model. It can be seen that the measured SPL shows a fast decline within the first 3 m and then tends to decline slowly. At the farthest end, the difference between the AEC model and experimental results is approximately 1.5 dB, and approximately 2 dB between the classical model and the experimental results.

To further demonstrate the model improvement, the predictive mean square error (MSE) of the original model and improved model is determined.

(11) $$\mathrm{MSE}=\frac{1}{n}\overset{n}{\underset{i=1}{\sum }}{\left(Y_{i}-\hat{Y_{i}}\right)}^{2}$$

where *n* is the number of samples, *Y_i_* is the real value, and $\hat{Y_{i}}$ is the predicted value. Here, the number of samples is 20 experimental results. Thus, the MSE can be obtained.

MSE (ADE model) = 3.394

MSE (AEC model) = 2.005

The MSE demonstrates a substantial improvement as the MSE difference between the ADE and AEC models is almost 1.3. It is evident that the improved model exhibits a noticeable enhancement in prediction accuracy.

## 6. Discussion

An “acoustic escape compensation” strategy for the acoustic diffusion equation model in underground long-channel-type spaces has been proposed. The strategy accounts for the transmission coefficient of the wall, which leads to the calculation of an SPL that is closer to the experimental results. A simulation and an experiment were performed to verify the proposed method, and before the underground experiment, an aboveground corridor experiment inside a building was tested to verify the applicability of the ADE model to long-channel-type spaces. The results show good agreement with expectations. It meets the expectation of improving the prediction accuracy without increasing the calculation time. The simulation time required for the four different compensation strategies mentioned in this article is approximately 0.7 s. Finally, the mean square error was introduced to evaluate the improvement effect, which shows a significant increase in the prediction accuracy.

The proposed strategy is derived from the transmission energy loss of coupled rooms, which improves the prediction accuracy. Although the improvement is not as big when compared to the experimental result of the field test, it provides a way to study the sound field of underground spaces and the advantages of the ADE model can still be utilized broadly in future applications.

Following this research, the reflection of sound particles in underground space is worth further study to perfect the prediction of SPL and support the management of underground spaces. The drawback of this method is that it is limited to applications in underground spaces since the relationship between the absorption coefficient and transmission coefficient is mutually constrained. The follow-up work of this study can focus on the impact of the AEC model on the prediction accuracy of reverberation time.

## 7. Conclusions

The purpose of this study is to explore the sound field characteristics of underground space. First, the difference in the acoustic characteristics of ground spaces and underground spaces is studied. Then, the superiority of the acoustic diffusion equation model with regards to the solving speed and the shortcomings in describing the acoustic characteristics of underground space is studied compared with other room acoustic modeling methods. The principle of the acoustic diffusion equation model is analyzed, and its shortcomings in describing underground space are noted. Moreover, the acoustic diffusion equation model is improved from the perspective of energy balance; that is, the energy “escaped” by the acoustic diffusion equation model in describing underground space is compensated. Simulation and comparative experiments are used to verify the proposed “acoustic escape compensation” model. The experimental results show that the proposed model has higher accuracy (mainly in the later stage of prediction) and smaller mean square error than the original model in predicting the sound field in underground space. This further verifies that the more closed special environment of underground space has a significant impact on acoustic propagation, and our proposed model can better describe the acoustic characteristics of underground space. However, we should also note the limitations of this study. For example, we only consider the sound field of a specific type of underground space according to our focus research area. Therefore, future research can be conducted to further expand our model to consider more types of underground space. We believe that this study provides a new perspective for the acoustic study of underground space and provides a valuable reference for future research.

## Figures and Tables

**Table 1:** SPL attenuation along the length at 10 m, 20 m, and 30 m.

SPL Attenuation (dB)	*τ* = 0	*τ* = 10^−5^	*τ* = 10^−4^	*τ* ≈ 3.2 × 10^−4^
x = 10 m	−16.62	−16.59	−16.34	−15.75
x = 20 m	−19.59	−19.53	−19.06	−18.08
x = 30 m	−21.33	−21.25	−20.57	−19.17

**Table 2:** The experimentally recorded data. Days 1, 2, and 3 represent 1, 2, and 4 June, respectively.

SPL	R1	R2	R3	R4	R5	R6	R7	R8	R9	R10	R11
day 1	91.2	88.4	87.4	87.1	86.8	86.5	86.1	85.7	85.2	85.0	84.8
day 2	90.9	88.1	87.4	87.1	86.6	86.3	85.9	85.8	85.3	85.1	84.7
day 3	91.3	88.0	87.2	86.9	86.6	86.5	85.9	85.6	85.4	85.0	84.7
Average	91.1	88.2	87.3	87.0	86.7	86.4	86.0	85.7	85.3	85.0	84.7
**SPL**	**R12**	**R13**	**R14**	**R15**	**R16**	**R17**	**R18**	**R19**	**R20**	**Red Point 2**	**Red Point 3**
day 1	84.4	84.2	83.6	83.1	82.7	82.3	82.3	82.1	81.9	57.5	52.8
day 2	84.1	83.8	83.3	82.8	82.6	82.6	82.4	82.2	82.2	57.2	53.1
day 3	84.5	84.1	83.6	83.3	82.9	82.4	82.4	82.1	82.0	57.4	52.9
Average	84.3	84.0	83.5	83.1	82.7	82.4	82.4	82.1	82.0	57.4	52.9

**Table 3:** The data required to calculate the SPL in cavity 1 and cavity 2 using the transmission method.

Parameter	Cavity 1	Cavity 2
*S*_12_	1.44	1.44
$\alpha_{a}$	0.02	0.02
*S*_2_	18.89	36.35
*A*_22_	0.38	0.73
log$\frac{S_{12}}{A_{22}}$	0.58	0.30
*R*	35.00	35.00
*L*_1_	86.40	84.00
*L*_2_ calculated	57.20	52.00
*L*_2_ measured	57.40	52.90

## Data Availability

Data will be made available upon request.
